# Mental health provision for children affected by war and armed conflicts

**DOI:** 10.1007/s00787-024-02492-w

**Published:** 2024-06-15

**Authors:** Panos Vostanis

**Affiliations:** 1 School of Media, Communication and Sociology, University of Leicester, University Road, Leicester, LE1 7RH UK; 2 Centre for Social Development in Africa, University of Johannesburg, Napier Road, Gauteng, Johannesburg, 2092 South Africa

## Status of evidence-base and emerging priorities

In recent years, there has been increasing public, media and policy attention on how children and young people may be affected by various war conflicts, and how their mental health and wellbeing can be supported in the short- and longer-term. As many as one in five (or 468 million) children globally live in zones of war or armed conflict [1]. Because the types and sociocultural contexts of these conflicts vary, emerging evidence on appropriate and effective interventions and service provision should be transferred, adapted and interpreted in respective post-conflict circumstances and systems. Overall, there is a strong body of epidemiological knowledge on prevalence of psychiatric disorders and broader psychosocial needs, complex mechanisms of risk factors involved and, to a lesser extent, protective factors that buffer war trauma and strengthen children’s resilience. Interventions were adapted from existing modalities or were developed by agencies on the ground before being tested, evaluated and scaled up. Policy and service guidelines were informed by this body of evidence, however, these were often not supported by robust research designs and findings. The aim of this paper is to synthesise theory, policy and research evidence in addressing certain gaps and priorities and highlighting areas for future growth.

The high prevalence of a range of child mental health problems in the aftermath of war and armed conflicts is well established, although prevalence rates vary significantly, because of different types and duration of trauma exposure, target populations and measures used. Overall, children are at elevated risk of suffering from post-traumatic stress, anxiety and depressive disorders, with a high degree of comorbidity, and continuity to young life and adulthood without available support [2, 3]. Identified risk factors (or vulnerabilities) are often inter-linked and can negatively impact on the child (direct and indirect exposure, cognitive capacity), family (loss of loved persons, prolonged separation, parental mental health and unemployment), and community (shortage of basic needs such as food and water, disrupted or damaged support networks, extended poverty, discrimination) [4]. These factors are compounded for unaccompanied minors, because of institutional care (orphanages), sexual or physical exploitation, and illegal labour [5]. Depending on the impact of war conflict on family, community and service systems, this can be mediated by adaptive coping strategies, self-efficacy, emotional regulation, positive parenting, forming and maintaining relationships, social networks, and access to informal or structural support [6, 7]. These findings, in conjunction with research on post-traumatic stress growth informed the design of interventions.

The aim of this paper is to critically evaluate the evidence from a child mental health service perspective across war conflicts, sociocultural contexts and systems, and to highlight implications for practice, service development, capacity-building and future research. Because of the uneven status of the literature [8], this is based on a narrative review of systematic and scoping reviews during the last ten years on the impact of war conflict and interventions for children and youth (referred to as children throughout the manuscript), with additional focus on service-related issues from the wider child mental health literature, where there was not sufficient evidence as yet for service systematic or scoping reviews per se. Although war conflicts and zones are heterogenous and not static, the paper is structured to broadly consider service provision in low- and middle-income (LMIC or Global South) countries, with brief additional reference to service principles for asylum-seeking and refugee children in high-income countries (HIC or Global North). Research findings and service recommendations should be understood within influential and intersecting conceptual frameworks, which are summarised and related to this field in the next section. Without an underpinning theory and model, interventions and services may otherwise use limited resources ineffectively, lack cultural sensitivity thus population engagement and uptake, and even carry the risk of re-traumatisation and harm [9].

## Conceptual frameworks

Children’s rights policy and legislation has influenced how their needs are viewed separately from those of adults, and how interventions and services are designed, usually in predominantly adult-centric systems [10]. Interestingly, war conflicts are increasingly viewed through children’s perspectives, and their safety has been extended to protecting their physical as well as psychological wellbeing. Research methods, measures and interventions are specifically designed for different developmental stages, including those of younger children, in promoting their participation [11] and in cross-cultural research [12]. Crucially, children’s rights should influence the child-centredness of environments and spaces during and post-conflict such as refugee camps, shelters and care homes. Although it is widely accepted by professionals that individual therapeutic interventions are non-effective or even contra-indicated until a child is removed from an abusive to a safe placement, the same principle is not as yet easily understood and translated when a whole child population cannot escape from ongoing war trauma exposure.

Such exposure impacts on children and their surrounding and inter-linked socioecological systems (individual, family, school, community, services and society) [13, 14]. Hence, interventions and services are more likely to be effective if these are multi-modal or multi-tiered targeting the child’s micro- and meso-system, while policy should also address macrosystems such as health, education, culture, law and geography [15, 16]. This approach is consistent with the conceptualization of resilience as “multi-level processes that systems engage in to obtain better-than-expected outcomes in the face or wake of adversity” [17, p.4], which has evolved from earlier understanding of resilience as an individual trait. Such a dynamic approach to strengthening the resilience of affected children and their systems is particularly important in war conflict contexts [18]. As traumatic memories and narratives and socioeconomic adversities can have a lasting effect on future generations [7], shared intergenerational and historical trauma and resilience [19] is increasingly included as another dynamically linked level [20]. Indeed, children were shown to draw on previous generations’ stories of resilience in relating to their own life circumstances, formulating new narratives and adaptive coping strategies [21]. Overall, interventions for war-affected children are also informed by cross-cultural and trauma-informed theories, which enable their generalisability in different settings [22].

Children’s complex mental health problems across their socioecology are reflected by service principles and models. In post-conflict resource-constrained settings, meeting their basic needs is a priority [23], while mental health provision is more likely to be effective if integrated to existing informal and structural psychosocial supports. The World Health Organisation [24] defines multiple levels of service integration, at micro- (person-focused, coordinated from end-user perspective); meso- (shared agency competencies, accountability, information systems); and macro-level (policy, funding, government structures). International bodies also endorse a stepped or scaled service model, irrespective of the extent of available resources, with levels of response that rationalise available community and professional skills and maximise impact [25, 26]. Capacity-building programmes should be designed in conjunction with such a service framework to be equipped for each level (or tier) of response, hence be provided in an interprofessional context through experiential learning [27, 28]. Training benefits are more likely to be sustainable if they adopt the Train-of-Trainer (ToT or cascade) approach that aims to upskill a core group of professionals, who then translate, transfer and cascade knowledge to other professional and community groups [29]. For this reason, it can maximise capacity and resources, even if specialists are scarce, thus enhance reach to large populations.

## Low- and middle-income countries: acute conflicts, internal or regional displacement

### Context, challenges and opportunities

The design and implementation of service provision in LMIC is faced with many generic and context-specific challenges. Overall, barriers to child mental health support include mental health concepts and stigma, parent engagement in competition with socioeconomic pressures, limited infrastructure and skilled workforce - especially specialist resources, and culturally sensitive interventions [30]. War conflicts, particularly if prolonged, place additional strain on basic needs, economy, employment and service systems. The main source of protection through informal family, peer and community networks can be disrupted or extensively damaged, as indeed schools, health and community centres. Children and parents are often re-traumatised and such exposure applies to professionals too. For example, Shamia et al. [31] found that post-traumatic stress disorders among health professionals was predicted by their war trauma exposure as civilians, rather than through their professional capacity. Internally displaced children and families may come from different ethnic groups to host communities, thus not being integrated, instead being exposed to discrimination and exclusion within their own or neighbouring country. As refugee camps and orphanages can be hastily set up during crises, without planning or adequate staffing, children are vulnerable to maltreatment and institutionalisation.

Nevertheless, collective societies offer opportunities to incorporate preventive and responsive interventions. Informal support networks through family units (including extended family), peers, communities and religious groups hold local knowledge, are trusted by children and parents, and constitute a resource for community volunteers [32]. Several studies in LMIC resource-constrained settings found that such networks are viewed positively by children, despite their lack of knowledge of structural services, and are crucial in initiating help-seeking [33]. Young people and parents can play a pivotal role as peer educators, mentors or support workers [21]. Humanitarian and development aid agencies, as well as community-based and religious organisations, already provide different levels of psychosocial support, although usually in silo, without clarity on their remit and related skills.

## Principles of service provision

Taking into consideration the extent of unmet need and limited resources, a service model should be designed at population level, albeit with stepped levels of care [34]. Interventions should largely be integrated, with a broad psychosocial remit, and delivered at schools, community centres, refugee camps or shelters. This requires close collaboration with humanitarian and recovery agencies on the ground, with agencies taking responsibility for their domain of expertise [15]. Inter-disciplinary working should be incentivised by joint policy and by donors through pooled budgets. Local stakeholders should be active in cultural adaptation and co-production of interventions and services, to enhance likelihood of uptake, retention and mobilisation [35].

Based on research findings, different response levels have been proposed for psychosocial interventions. In this model of provision, we consider three levels through awareness and psychoeducation, resilience-building, and therapeutic ‘recovery’ or re-processing trauma. It is acknowledged that interventions may overlap across response levels, because of fluid conflict conditions and population movement. For the first response level, all children should be considered as vulnerable in post-conflict situations. Implemented psychoeducation and first aid programmes include symptom management, mindfulness, guided imagery, life skills training, emotional regulation, self-expression and problem-solving [36, 37]. These were found to variably improve wellbeing, social and trauma-related functioning and, to a lesser extent, mental health symptoms [38, 39].

The second response level could target at risk groups, by means of proximity, loss, displacement or emerging mental health problems. A third specialist response level is desirable for children with severe disorders, although the few available mental health professionals should consider outreach clinics, group interventions, consultation and training to frontline agencies, as essential components of their workload. Delineation between response levels 2 and 3 is not always clear in the literature, however, moderate positive effects on mental health symptoms were reported for trauma-focused cognitive-behavioural and narrative exposure therapies, with less conclusive evidence on widely used creative expressive therapies [36]. Interestingly, common change mechanisms were identified across modalities such as cognitive restructuring, trauma re-processing, rapport-building, strengthening relations with caregivers, relapse prevention, family and community capacity-building [37, 40].

A major issue for caution and debate is the extent and nature of interventions in the face of continuing conflict. Emerging evidence indicates that interventions should not be withheld during ongoing threat, with positive findings on children’s mental health [41]. However, these should not compensate for safety, hence be different to therapeutic interventions planned in relative stability, instead focusing on safety planning, a degree of internal security, and strengthening physical and emotional resources, thus building resilience at that stage [16]. This is particularly important for interventions delivered by non-specialists, who can delve into trauma re-processing instead of resilience-building. Training and supervision should thus also focus on knowledge and competencies in ‘closing’ instead of ‘opening’ trauma-related conversations with children and parents. Community volunteers were shown able to acquire skills and deliver first level response [39, 41]. However, they need to be integrated to a comprehensive service model and receive ongoing training and supervision. Parallel attention should be paid to providing child-centred environments and protective infrastructures of internal ‘safe’ havens for children. Different contexts may require targeting the specific needs of groups such as previous child soldiers, orphaned children or victims of kidnapping, as well as accounting for gender issues [42, 43].

**Fig. 1:**
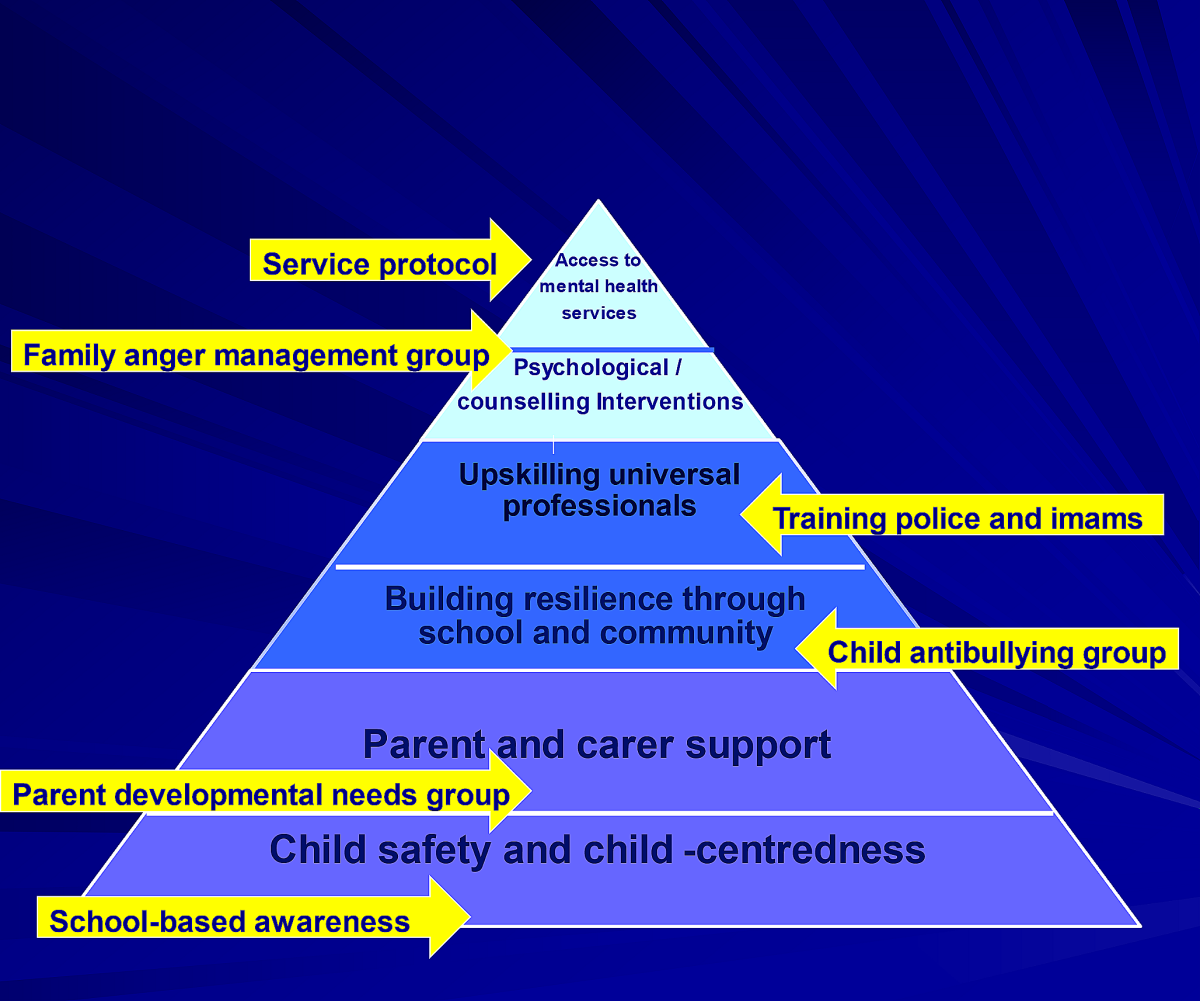
Multi-modal service framework for refugee children in Turkey

Based on the stepped-care service model and previously discussed theories, several frameworks of service provision have been put forward [25]. The Inter-Agency Standing Committee guidelines on mental health and psychosocial support in emergency settings [44] proposed four tiers of basic services and security, family and community support, non-specialist and specialist support. Taking into consideration the emerging evidence-base in LMIC resource-constrained settings, we extended this multi-modal service framework to six inter-linked domains of protection and child-centredness, family and caregiver support, building resilience through school and community, upskilling universal practitioners and community volunteers, counselling and psychological interventions, and access to mental health services [45]. Stakeholders can apply the framework in formulating service plans across the six domains. This framework was co-designed, piloted and evaluated in several LMIC, including with refugee children and families [46, 47]. Figure 1 below illustrates an example of co-producing service plans for refugee children in Turkey. Such a framework can be combined with capacity-building through the Train-of-Trainer approach, thus ensure sustainability [48].

## High-income countries: forced migration

### Context, challenges and opportunities

Most evidence children’s mental health needs in high-income settings involves mixed child populations seeking asylum or undergoing resettlement in the host country, with limited research on the migration journey or children in transit between HIC. Prevalence rates remain high and needs are largely unmet, because of several barriers [2]. End-user barriers include mental health concepts and stigma, lack of information, prioritisation of basic needs, language difficulties, legal uncertainty, and distrust in host country authorities [49]. Systemic challenges are related to lack of care pathways, agency fragmentation, and insufficient contextualised professional skills [8]. Despite the higher level of resources, HIC service systems are not necessarily designed or equipped to respond to children affected by war trauma, especially when entry numbers rise. Interestingly, in a comparative study of support systems between Turkey and the UK, Eruyar et al. [50] established that, despite structural differences between the two systems, barriers to accessing support in both countries included stigma, shame, language acquisition and agency fragmentation.

Because of lack of designated care pathways, refugee children cannot access mainstream services through the usual routes of primary health, social care or schools, thus often come in contact with mental health professionals when in crisis, thus resulting in disproportionate and often involuntary in-patient admissions, but without after-care community plans [51]. Despite the availability of specialist skills, professionals may not be familiar with the particular needs of refugee children, thus trained in cross-cultural and trauma-informed interventions. Staff in community and residential settings such as shelters often lack training in the integration of mental health approaches [52]. Care systems vary considerably for unaccompanied minors, in relation to protection and psychosocial support [53].

## Principles of service provision

Policy, services and interventions need to address refugee children’s complex needs, lack of access and engagement, transition, stigma, legal uncertainty, and cultural characteristics [54]. Joined up policy and commissioning will encourage inter-agency working on the ground. Care pathways should be joint and direct, with referrals being processed by a local inter-agency network that will ensure collaborative care and efficient use of resources [8]. Although the principles and frameworks of therapeutic modalities would not largely differ from those described in the previous LMIC section, there will be more specialist choices and availability of psychodynamic, creative or targeted interventions such as EMDR, as well as pharmacological treatment where appropriate [4, 55]. Overall, multi-modal programmes and services have been shown to offer comprehensive and sustained care [56]. A designated service may not be realistic, even in urban areas with large numbers of refugee children. However, its principles could be adopted in conjunction with other vulnerable groups such as children in public care, or by partially allocating designated sessions and clinics to mental health professionals. They would thus be able to attend inter-agency meetings, and provide outreach work, consultation and training.

Capacity-building is important in improving the quality of everyday psychosocial care and relieving unnecessary pressures on specialist services. Community and residential workers may have practical experience in supporting refugee children but lack sufficient mental health knowledge and skills within their role [52]. For this reason, training should both be contextualised and strategic, as part of national or local initiatives. For example, a Train-of-Trainer programme across all shelters for unaccompanied minors in Greece established a trainer pool of mental health professionals, who then translated and transferred knowledge to their local staff base, including caregivers, cooks and administrators who spent most time with the children. Trained staff identified important training ingredients of relating to their remit, addressing emotional impact of trauma on themselves as well as the children, interprofessional learning and experiential approaches, but also cautioned that training required organisational support to be sustainable [57].

## Conclusion and future directions

The huge number of children affected by war and armed conflict globally pose a serious challenge for humanitarian and other support organisations and agencies globally. On a positive note, there is increasing recognition and emerging evidence-base on their mental health needs and indications for preventive and responsive interventions in various post-conflict contexts, as well as during continuing threat. This is an important opportunity for child mental health professionals to make a difference by integrating mental health to psychosocial care, diversifying their skills across cultures and systems, and offering consultation and contextualised training to frontline practitioners and community volunteers. Initiatives need to be promoted and supported by international organisations, governments and professional bodies through joined-up policy, guidelines, pooled budgets, international networks and trainer capacity.

## Data Availability

No datasets were generated or analysed during the current study.
